# Mesh reconstruction of the myopectineal region following wide local excision for inguinofemoral tumors

**DOI:** 10.3389/jaws.2026.16648

**Published:** 2026-07-27

**Authors:** Paul Yohan George, Paul Trinity Stephen, D. K. Titus, Beulah Roopavathana, Nitin Paul Ambrose, Prakash Joseph, Suchita Chase

**Affiliations:** Surgery Unit 4 (Sarcoma and Abdominal Wall Reconstruction Unit), Christian Medical College and Hospital, Vellore, India

**Keywords:** extraperitoneal mesh reconstruction, groin reconstruction, incisional hernia prevention, inguino-femoral tumour excision, myopectineal region reconstruction, soft tissue sarcoma

## Abstract

**Introduction:**

Appropriate reconstruction following radical resection of sarcomas in the inguinofemoral region and the pelvis is essential to prevent the subsequent development of an incisional hernia. Simple soft-tissue coverage may not suffice, as resection often involves the removal of the flat muscles and the inguinal ligament. We propose a technique of extraperitoneal mesh placement to reconstruct the myopectineal orifice (MPO).

**Methods:**

In this paper, we present a technique of extraperitoneal mesh reconstruction of the myopectineal region and inguinal ligament following radical resection for inguinofemoral tumours. The short-term surgical outcome, long-term oncological outcomes, and the development of an incisional hernia were also retrospectively reviewed.

**Results:**

A total of 13 patients were included in the study from May 2017 to February 2025. In 11/13 patients, primary closure of the soft tissue defect was possible. In two patients, a local rotational flap was required for soft tissue coverage. In 11/13 patients, a polypropylene mesh was used. In two patients, a titanium-coated polypropylene mesh was used. For all patients, the mesh was placed in the extra-peritoneal plane. Regarding short-term complications, 4 patients developed a surgical site infection, and 2 developed a seroma. In terms of long term outcomes, only one patient was confirmed to have developed an incisional hernia.

**Conclusion:**

This technique offers a simple, safe, cost-effective, and reproducible method for reconstructing the myopectineal orifice in patients undergoing radical resection of soft tissue tumors involving the inguinofemoral region, with the sacrifice of the inguinal ligament.

## Introduction

Management of soft tissue tumors involving the inguinofemoral, iliopsoas, or pelvic region can often be challenging. Radical resection in this region, besides leaving a significant soft-tissue defect, often involves transecting the inguinal ligament, thereby opening the various spaces of the myopectineal orifice, and putting the patient at risk of developing a hernia.

Several options exist for soft tissue reconstruction, such as local and free flaps [1–3]. However, techniques for reconstructing the inguinal ligament and myopectineal region are sparse in literature.

In this paper, we describe a series of patients in whom we used a mesh to reinforce the myopectineal region and reconstruct the inguinal ligament.

## Materials and methods

### Ethics

Written informed consent was obtained from the individual for the publication of any potentially identifiable images or data included in this article.

### Methodology

We retrospectively reviewed a series of 14 patients who had undergone a radical resection of a soft tissue tumor in the inguinofemoral, iliopsoas, or pelvic region, which involved resection of the inguinal ligament and reconstruction with the described technique. One out of the 14 patients was excluded from the final analysis as a polyglactin mesh was used. The mesh being rapidly absorbable would inherently put the patient at an increased risk of developing an incisional hernia as compared to a polypropylene mesh and would confound the results. To maintain the homogeneity of the cohort, this patient was excluded from the final outcome analysis.

The data collected included patient demographics, oncological details like histopathology of the tumor, staging, location, and outcomes in terms of post-operative complications, local tumor recurrence, and development of an incisional hernia.

Long-term follow-up data, such as local recurrence or development of an incisional hernia, were collected either based on records from a recent outpatient visit or by telephonic enquiry.

The study was conducted in a tertiary care center in south India. Data was collected between the years 2017–2025.

### Description of the technique

**FIGURE 1:**
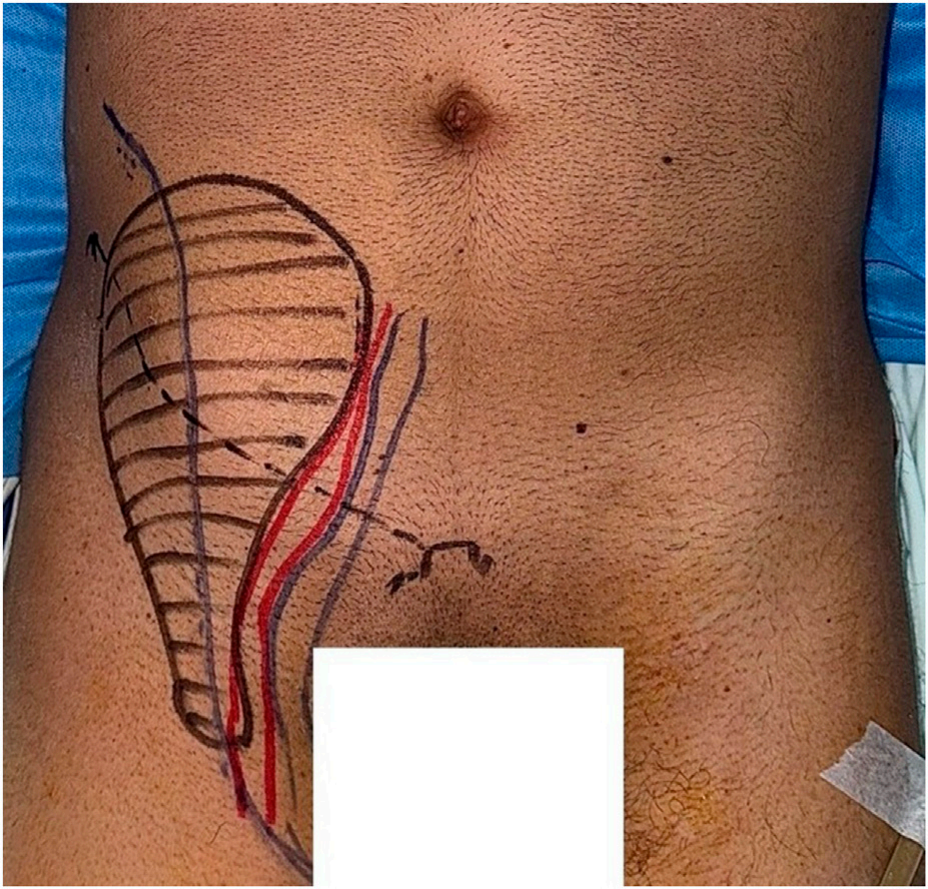
Tumor with borders marked.

**FIGURE 2:**
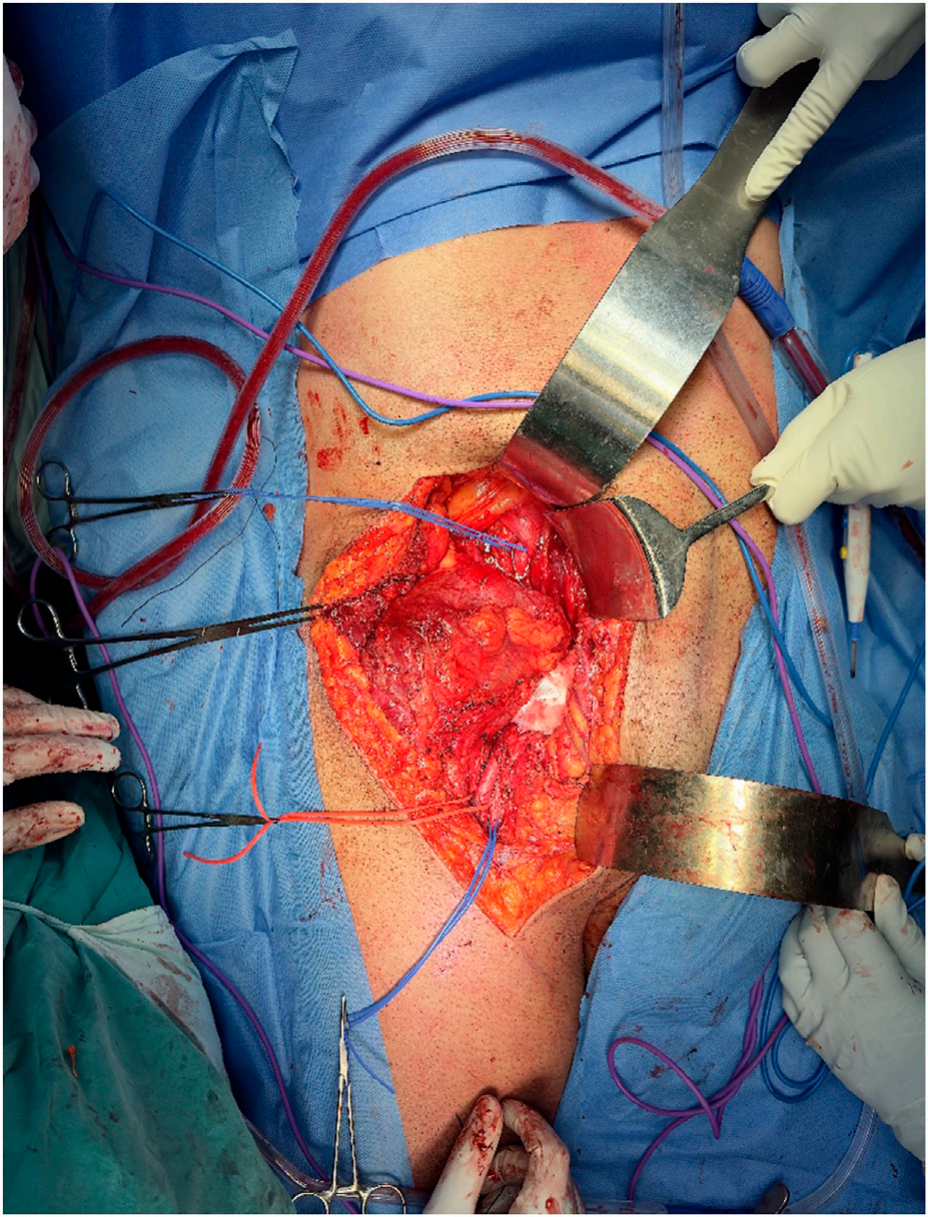
Tumor-intra op.

**FIGURE 3:**
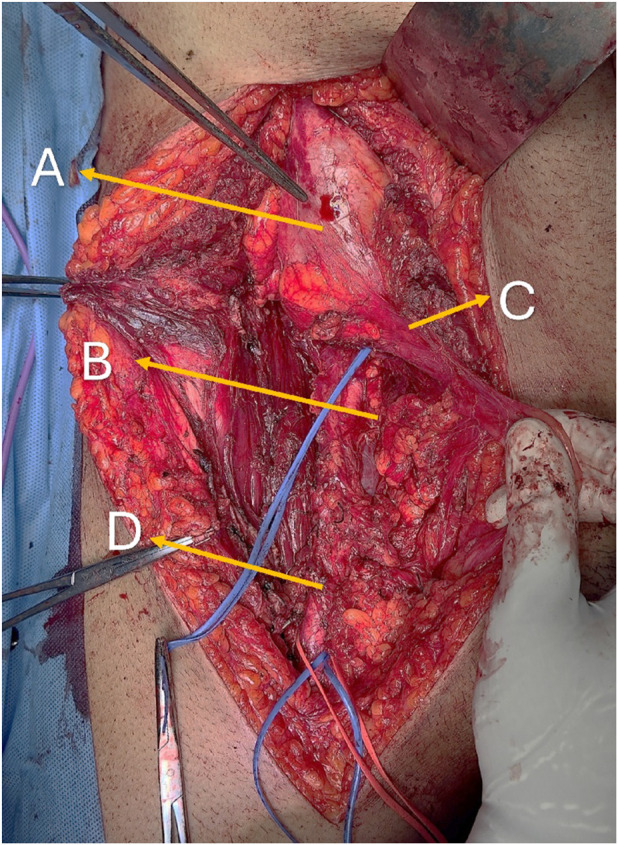
Tumour bed-post excision. Neurovascular structures were preserved, but inguinal ligament was excised en bloc with the mass. Picture shows the Peritoneum kept intact (A), The External iliac vein (B), The gonadal vessels (C) and femoral artery (D).

Soft tissue tumors involving the inguinal region were excised *en bloc* with adequate oncologic margins (Figures 1–3). Superior to the inguinal ligament, the dissection was maintained in an extraperitoneal plane, and any inadvertent peritoneal breach was repaired primarily. This excision resulted in a composite defect of the lower abdominal wall with loss of the abdominal wall musculature and inguinal ligament (Figure 3).

**FIGURE 4:**
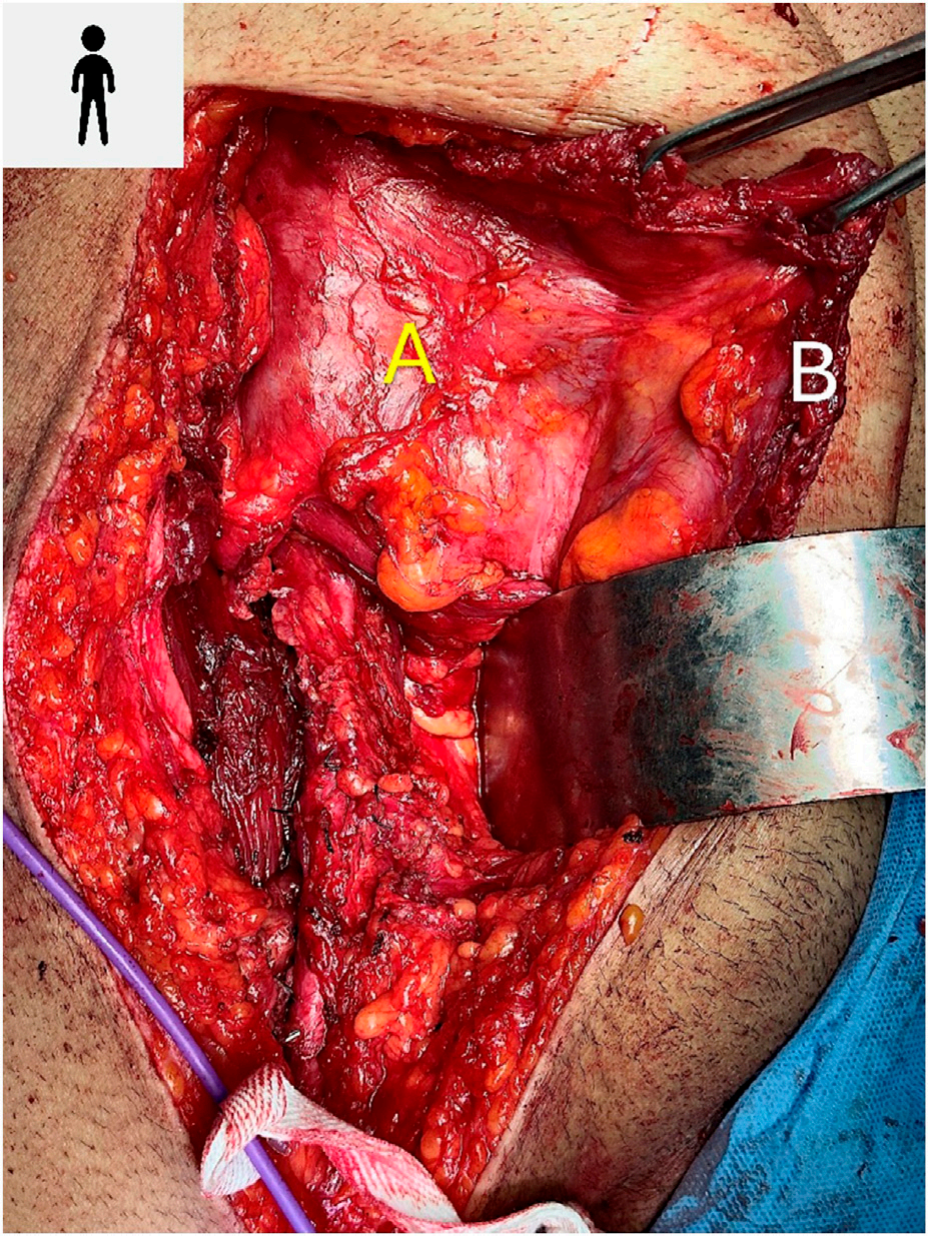
Creating the pre peritoneal space - A-peritoneum, B- flat muscles.

**FIGURE 5:**
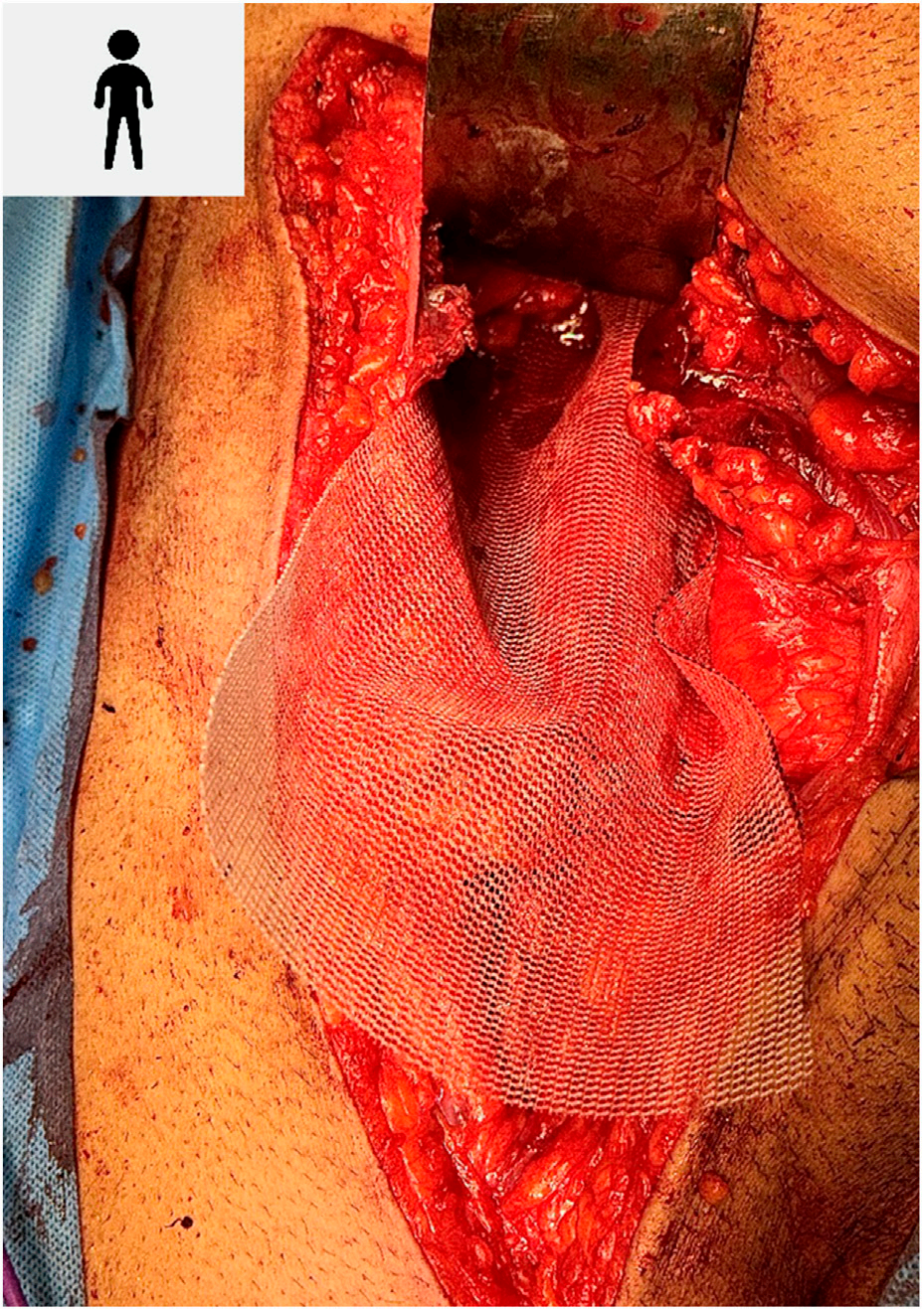
Inferolateral edge of mesh placed pre-peritoneally. Polypropylene mesh of appropriate size is placed and anchored posteriorly from lateral abdominal wall to the edge of vertebra using tackers without injuring the neuro vascular bundle.

**FIGURE 6:**
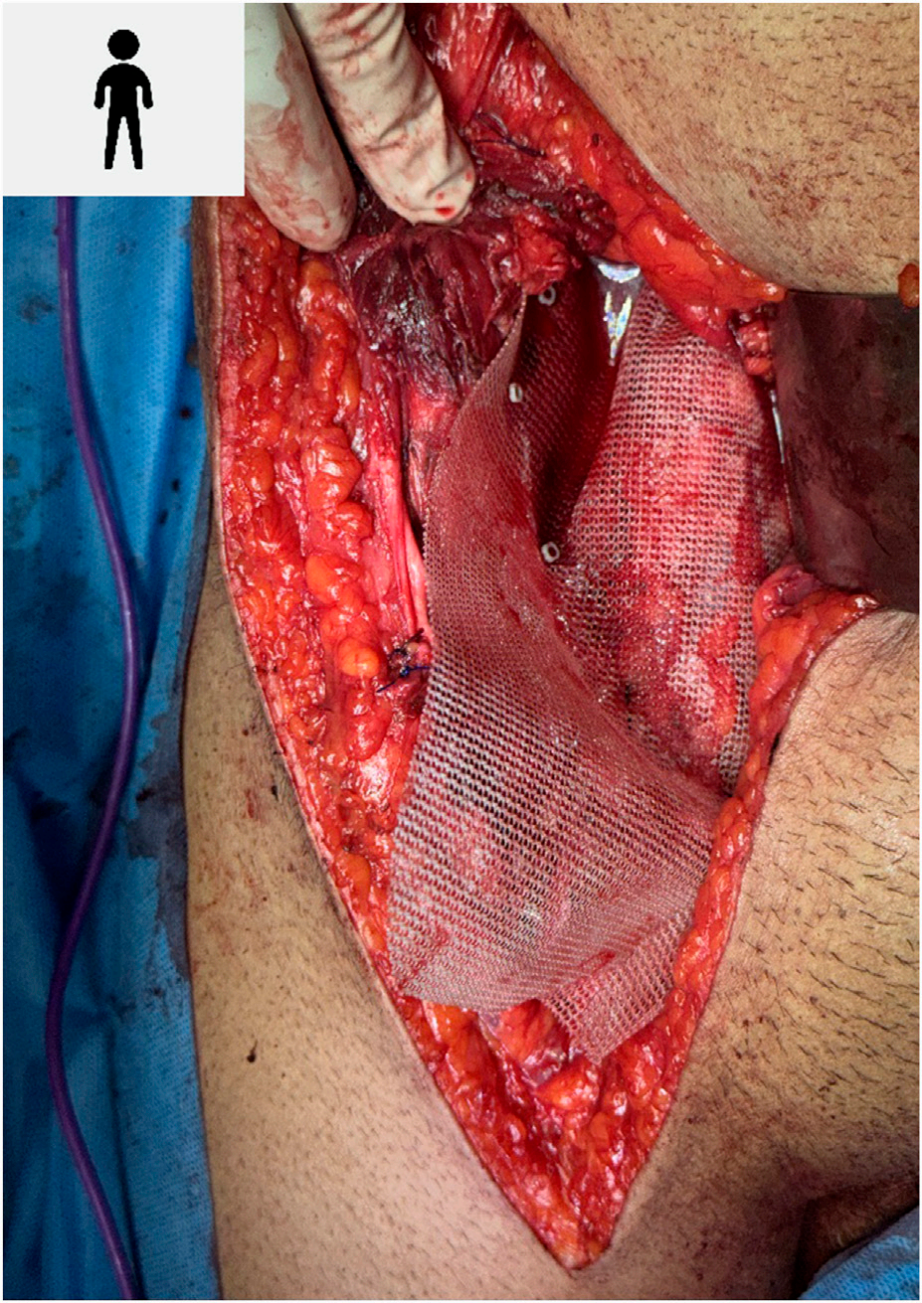
Inferolatral edge of mesh fixed to ilio-psoas. Polypropylene mesh of appropriate size is placed and anchored posteriorly from lateral abdominal wall to the edge of vertebra using tackers without injuring the neuro vascular bundle.

Reconstruction of this defect was performed using a prosthetic mesh placed in the extraperitoneal space (Figure 4), configured in a hammock-like manner. Superiorly, the mesh (commonly polypropelene) was anchored to the parietal abdominal wall using transfascial sutures or tackers. Inferiorly the mesh was secured medially to Cooper’s ligament, folded downward, and its edges were anchored sequentially to the psoas, the iliacus and the anterior superior iliac spine. Laterally, the mesh was tucked and fixed to the transverse process of the vertebra, taking care to preserve the neurovascular bundle (Figures 5, 6).

**FIGURE 7:**
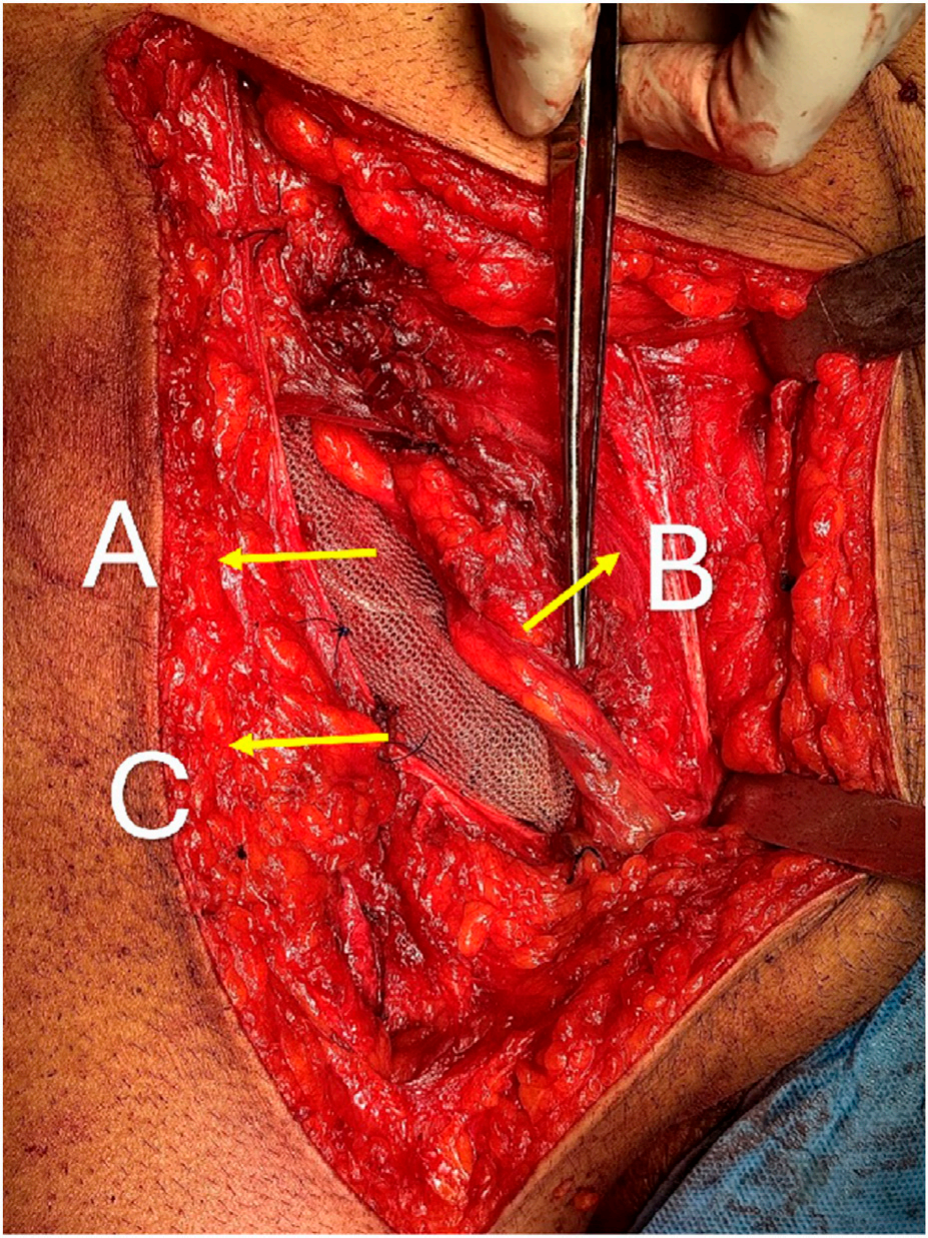
Final mesh configuration, male patient. Mesh folded on itself and tucked in the pre peritoneal space (A). Cord structures medialized and exiting through superficial ring (B). Inguinal ligament reconstructed if possible (C). Folded portion of mesh acts as a neo inguinal ligament (D) or reinforced the reconstructed ligament (C).

**FIGURE 8:**
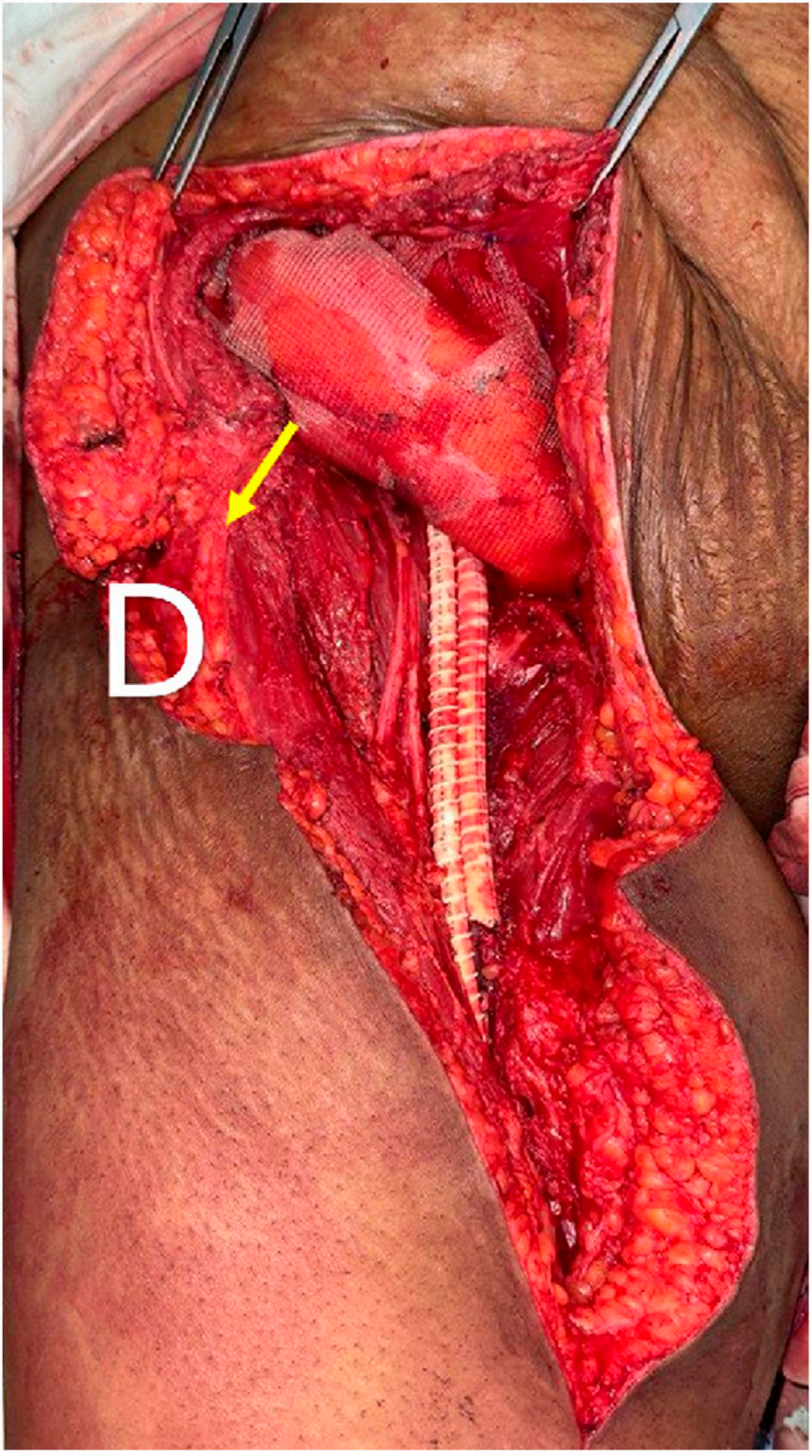
Final mesh configuration, female patient. Mesh folded on itself and tucked in the pre peritoneal space (a). Cord structures medialized and exiting through superficial ring (b). Inguinal ligament reconstructed if possible (c). Folded portion of mesh acts as a neo inguinal ligament (D) or reinforced the reconstructed ligament (c).

The folded edge of the mesh effectively served as a reconstructed inguinal ligament. In male patients, the testicular vessels are medialized and directed into the scrotum along with vas deference or a new deep ring was created by fish-tailing the lateral end of the mesh (Figures 7, 8). Finally, the residual anterior abdominal wall musculature was closed in layers or reconstructed with flap, and skin closure was performed as appropriate.

## Results

A total of 13 patients were included in the final outcome analysis (one was excluded as a polyglactin mesh was used). Patient demographics, tumor characteristics, and details of the reconstruction are summarized in Table 1. Thirty -day morbidity is summarized in Table 2 and Oncological outcomes are provided in Table 3.

**TABLE 1:** Summary of demographics, surgical technique and post operative outcomes.

N = 13	Number of cases
Demographics	
Male	9
Female	4
Age (Mean; SD)	38.3 (+/- 14.8)yrs
Maximum tumor dimension (mean; SD))	12.3 (+/- 6.07)cm
Tumor location	
Iliopsoas	5
Inguino-femoral region	4
Pelvic	4
Tumor histology	
Chondrosarcoma	3
Synovial sarcoma	4
Malignant melanoma	1
De-differentiated liposarcoma	1
Epitheloid sarcoma	1
High grade pleomorphic sarcoma	1
Undifferentiated	2
Surgical technique	
Surgical approach	
Extraperitoneal	10
Transperitoneal	3
Soft tissue reconstruction	
Pedicle rotation rectus abdominis myocutaneous flap	1
Tensor fascia lata rotation flap	1
Primary closure	11
Vascular reconstruction	3
Mesh characteristics	
Type of mesh	
Ti mesh	2
Polypropylene mesh	11
Mesh size	
15 × 15 cm	6
30 × 30 cm	5
23 × 20	1
25 × 20	1
Post operative complication	
SSI	4
Seroma	2
Incisional hernia	1

**TABLE 2:** 30 days morbidity.

S. No	Morbidity (CDG grade)	Treatment
1	SSI with superficial wound dehiscence (CDG 1)	Daily dressings-healed by secondary intention
2	Seroma (CDG1)	Managed conservatively- resolved
3	SSI (CDG 1)	Antibiotics and suture line negative pressure wound dressing
4	SSI (CDG 1)	Daily dressings-healed by secondary intention
5	SSI with superficial wound dehiscence (CDG 1)	Negative pressure dressing followed by skin graft
6	Seroma (CDG 3a)	Ultrasound guided drain insertion

**TABLE 3:** Oncological outcomes.

Margin status	
Negative	5
Close	6
Positive	2
Follow up	
Lost to follow up	2
Telephonic follow up	7
Out-patient follow up	4
Median follow up (month, IQR)	12 (7–24)
Outcomes	
Mortality (all cause)	4
Local recurrence	3
Distant metastasis	1

### Surgical approach

An extraperitoneal approach was employed in the majority of patients (10 of 13) to achieve tumor access and resection. Two patients required hemipelvectomy to obtain adequate oncologic margins due to the tumors origin from the bony pelvis. In all cases, prosthetic reinforcement was performed using a mesh placed in the extraperitoneal plane.

The primary material used for reconstruction was a polypropylene mesh, applied in 11 of 13 patients. A Titanium-coated polypropylene mesh was used in two patients. In the first case requiring a titanium-coated mesh, the patient had a markedly attenuated peritoneum with multiple tears that required primary repair, prompting the use of a mesh with a more bowel-friendly interface. In the second case, extensive sub-adventitial dissection around major vessels necessitated avoiding direct mesh–vessel contact; hence, a titanium-coated mesh was chosen for its inert, biocompatible surface.

We would also like to draw the readers’ attention to one patient in whom a polyglactin (Vicryl) mesh was used because an inadvertent bladder injury contaminated the field. This patient was excluded from the study cohort to maintain homogeneity, as a polyglactin mesh is a rapidly absorbable material and its use inherently confers a higher risk of subsequent incisional hernia formation. In such situations, the authors would instead recommend the use of a poly-4-hydroxybutyrate (P4HB) mesh, which has been shown to be a relatively safe option in contaminated fields and provides long term reinforcement while maintaining acceptable outcomes [4, 5]. Unfortunately, a P4HB mesh was not available at our institution at the time this patient underwent surgery.

Mesh dimensions varied across cases and are summarized in Table 1. Primary closure of the abdominal wall defect was achieved in most patients (11 of 13), while two required rotational flap reconstruction. Vascular reconstruction was undertaken in three patients.

### Margin status

Wide local excision aimed at achieving R0 resection was attempted in all patients. Among these, 6 patients had at least one close margin (<0.1 cm), 2 patients had a positive margin, and 5 patients had clear (negative) margins.

### Postoperative outcomes

Within 30 days post-surgery, 4 patients developed surgical site infections. Among these, 2 experienced complete to near complete superficial wound dehiscence. Importantly, none of the patients with wound dehiscence had exposure of the mesh or a mesh infection.

Two of these patients were managed with daily dressings, and the wounds healed by secondary intention. Two of these patients were managed with negative pressure wound therapy, of which one eventually required a split-thickness skin graft to cover the wound.

Seroma formation occurred in 2 patients. Management included conservative management with empirical antibiotic therapy in one patient and ultrasound-guided drain placement in another. Both patients improved.

The details of postoperative outcomes and treatment are summarized in the Table 2.

The average length of hospital stay was 17 days (range: 9–32 days).

There were no surgery-related mortalities and no documented cases of mesh infection.

### Follow-up and long term outcomes

At the time of analysis, four patients had a documented visit to the outpatient department at least a year postoperatively, and seven were contacted telephonically. Two patient were not contactable. The median follow up period was 12 months with an interquartile range of 7–24 m.

Among these patients, three had developed a local recurrence and one had developed distant metastasis with no local recurrence. Four patients had passed away during the follow-up period, including two who experienced early recurrence within 6 months of surgery.

One patient developed an incisional hernia, which was asymptomatic and picked up only on surveillance imaging.

With regard to quality of life, a formal assessment was not done, however none of the patients (those who had local recurrence were excluded from this particular assessment to avoid confounding)complained of debilitating chronic pain and were able to carry out their activities of daily living.

Patient-specific oncological outcomes are detailed in Table 3.

## Discussion

The management of soft tissue tumors involving the groin often necessitates a multidisciplinary approach due to the frequent involvement of multiple anatomical compartments. Surgical resection may require removal of skin, muscle, bone, and in some cases, vascular structures. While several options exist for soft tissue reconstruction, *en bloc* resection of the inguinal ligament disrupts the structural integrity of the myopectineal orifice, significantly increasing the risk of postoperative hernia formation.

Currently, no standardized technique exists for reconstructing the inguinal ligament. Various methods have been described in the literature, as summarized in Table 4. Alasdair et al. [6] reported using a tensor fascia lata flap to reconstruct the inguinal ligament in the context of a large recurrent hernia. Offodile et al. [7] described reconstruction in a series of six patients with pelvic malignancies using an AlloDerm-polypropylene composite mesh anchored to the anterior superior iliac spine (ASIS) and pubic tubercle via bone anchors. Similarly, Sama et al [8]. employed a technique in which the mesh was rolled into a cylindrical configuration and fixed to the ASIS and pubic tubercle, forming a “neo-inguinal ligament.”

**TABLE 4:** Review of literature.

Author	Year	Sample size	Technique	Follow up (median)	Hernia
Offodile et al.	2015	6	Alloderm prolene composite mesh fixed across defect with bone anchors	25.8 m	No hernia
Alasdair R. Bott et al.	2013	1	Pedicle fascia lata falp to reconstruct inguinal ligament	9 m	No hernia
V. Louis et al.	2023	7	Hammock mesh reconstruction	17.8 m	No hernia
L. Sama et al. (only abstract available)	2024	7	Cigarette rolled mesh between asis and pubic tubercle	Not mentioned	No hernia

While these techniques provide viable options for recreating the inguinal ligament, they often do not address the reconstruction of the anterior wall of the myopectineal orifice, which is commonly resected during extensive oncologic procedures. Louis et al. [9] described a “hammock-style” mesh placement closely resembling our technique. However, key differences exist: most tumors were approached via a transperitoneal route in their series, whereas our approach was predominantly extraperitoneal. Additionally, their reconstruction used Phasix™ mesh exclusively, whereas in our series, a heavyweight polypropylene mesh was preferred, except in specific clinical situations that necessitated alternative materials.

Our technique offers several advantages:

1. Extraperitoneal Mesh Placement: This avoids direct contact with intra-abdominal contents, reducing the risk of bowel adhesions and mesh-related complications.
2. Reconstruction of a Neo-Inguinal Ligament: The folded mesh is anchored securely to the ASIS and pubic tubercle, effectively replicating the structural function of the native ligament.
3. Comprehensive Defect Coverage: The mesh reinforces the entire myopectineal region, offering robust support to both primary and flap reconstructions.
4. Cost-effective: Placement of the mesh in the extraperitoneal space allows for the use of a polypropylene mesh, which is more cost-efficient in low-resource settings.

### Limitations

Despite these benefits, certain limitations must be acknowledged. The study’s small sample size and retrospective design limit the generalizability of the findings. However, larger prospective studies are inherently challenging given the rarity of these tumors. Additionally, follow-up was predominantly conducted via telephone interviews. While only one patient was diagnosed with an incisional hernia, the absence of routine clinical examinations and imaging limits definitive conclusions regarding the long-term incidence of incisional hernias.

### Conclusion

This technique offers a simple, safe, cost-effective, and reproducible method for reconstructing the myopectineal orifice in patients undergoing radical resection of soft tissue tumors involving the inguinofemoral region, with the sacrifice of the inguinal ligament. Though, due the nature of the disease it self, local recurrence may be high, the patients quality of life may be improved by the use of this technique to avoid the development of an incisional hernia.

Further studies with longer-term clinical and radiological follow-up are warranted to validate the durability and efficacy of this approach.

## Data Availability

The raw data supporting the conclusions of this article will be made available by the authors, without undue reservation.
